# Supplementary material for: Association between oral microbiome and seven types of cancers in East Asian population: a two-sample Mendelian randomization analysis
Source: Front Mol Biosci. 2023 Nov 21;10:1327893. doi: 10.3389/fmolb.2023.1327893 (PMC10702768; doi:10.3389/fmolb.2023.1327893)
Supplement: Supplementary file 1 [file DataSheet1.ZIP › Supplementary Materials/MR plots of saliva/saliva═╝/Prostate cancer/pheno.2938_to_prostate cancer_forest.pdf]

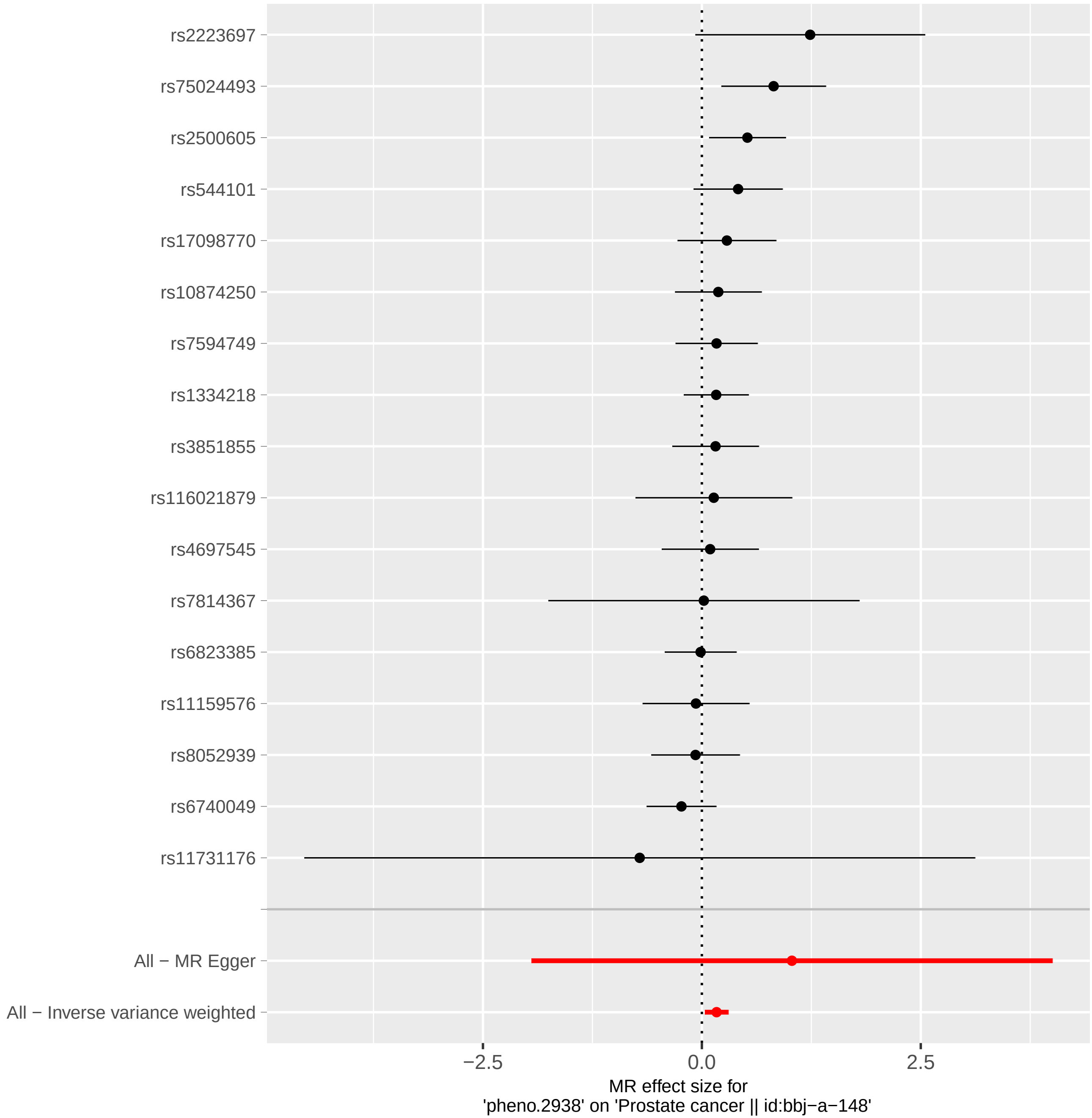
rs2223697
rs75024493
rs2500605
rs544101
rs17098770
rs10874250
rs7594749
rs1334218
rs3851855
rs116021879
rs4697545
rs7814367
rs6823385
rs11159576
rs8052939
rs6740049
rs11731176
All − MR Egger
All − Inverse variance weighted
−2.5
0.0
2.5
MR effect size for
'pheno.2938' on 'Prostate cancer || id:bbj−a−148'
